# KRAS and TP53 Mutations in Colorectal Carcinoma

**DOI:** 10.4103/1319-3767.56087

**Published:** 2009-10

**Authors:** Khawla S. Al-Kuraya

**Affiliations:** Human Cancer Genomic Research, Research Center, Department of Colorectal Surgery, King Faisal Specialist Hospital and Research Centre, Riyadh, Saudi Arabia

Colorectal cancer is a major cause of mortality and morbidity worldwide. However, various therapeutic modalities followed in clinical practice are not life saving. More recently, advances in understanding tumor biology have led to development of targeted therapies[1] allowing progress in the treatment of colorectal cancer.[2] Colorectal carcinogenesis is characterized by numerous genetic and epigenetic changes including mutations of oncogenes, tumor suppressor genes and mismatch repair genes, microsatellite instability, allelic losses in specific chromosomal arms, and methylation changes in gene promoters.[3] Colorectal carcinoma (CRC) consists of two subtypes; the chromosomal instability (CIN) subtype, associated with loss of function of the tumor suppressor gene *TP53*[4] and microsatellite instability (MSI) subtype resulting in accumulation of mutations, accounting for approximately 10-20 % of the cases, characterized by inactivation of the mismatch repair (MMR) proteins.[5]

According to the Fearon and Vogelstein colon cancer model, APC (Adenomatous Polyposis Coli) gene is involved in adenoma formation and KRAS oncogene in the transition from intermediate adenomas to carcinomas in sporadic CRC.[6] Thus, somatic *KRAS* mutation is an early event in colorectal carcinogenesis, predominantly occurring during the transformation of a small to intermediate sized adenoma.[7] In this issue of Saudi Journal Of Gatroenterology, Sameer and colleagues have studied p53 and K*RAS* mutations in colorectal carcinomas in a unique ethnic Kashmiri population from India.[8] They have found p53 and *KRAS* mutations to be common genetic changes and are implicating p53 mutations to be the predominant risk factor in Kashmiri CRC.

In CRCs, *TP53* gene mutations and allelic loss on 17p are genomic alterations that occur as late events in tumor progression. According to published reports, *TP53* mutations have been described in about 40-50% of colorectal cancer cases.[9] *TP53* gene mutation incidence in colon cancer from Saudi Arabia is 33.7 %.[10] This is much lower than that reported in the West. Similar prevalence figures of *TP53* mutations (32.3 %) have been reported in Eastern Europe.[9] Apart from possible ethnic differences causing differences in incidences, there is conflicting evidence on the prognostic significance of *TP53* gene mutations expressed in colorectal carcinomas.[11]

Majority of the TP53 mutations occur in the core domain (exons 5 to 8) which code for residues 130-286, the most important region responsible for folding and therefore, for stabilization of the tertiary structure of the protein. These mutations result in the loss of p53-binding ability to DNA and eventually its function. Sameer *et al*, screened for *TP53* mutations in Kashmiri CRC patients; the incidence of *TP53* mutations was 45%.[8] Further analysis of these *TP53* mutated cases also revealed the high percentage of G: C > A: T (53.57%) and G: C > C: G (14.28%) transition and transversion mutations respectively. A peculiarity of *TP53* mutation in Kashmiri CRC tumors was the lack of deletion (0 *vs.* 6.57% in IARC) and a higher prevalence of insertion (17.85 *vs.* 1.4 % in IARC R12, release). The high prevalence of G: C →A: T mutation was again an observation of interest in the study. The presence of alkyl nitrosamine in foodstuffs, leading to O^6^-alkyl guanine adducts and base misrepairing during replication, resulting in G→A transition has been considered a major risk factor in China. However, establishing a correlation between the enhanced G→A transition (53.56%) in their study and the presence of nitrosamines in the foodstuffs used in Kashmir[12] needs further investigation. G: C→C: G transition was comparatively high in Kashmiri samples (14.28 *vs*. 7.51% in IARC). The G: C→T: A transversion was observed to be confined to males who smoked.

Sameer and colleagues reported the *KRAS* mutation incidence of of 22.6 % in CRC from Kashmir. *RAS* genes, for the past 30 years or so, have always been at the leading edge of signal transduction and molecular oncology.[8] However, since their discovery in acute transforming viruses to current post genomic era, a complete understanding of RAS function and dysfunction, mainly in human cancer, is still to come.

The three classical mammalian *RAS* genes, K-, N- and H *RAS*, encode 21 kDa proteins that are members of the guanine nucleotide binding protein super family.[13] Ras proteins function as plasma membrane-bound guanine nucleotide binding proteins with intrinsic GTPase activity and act as molecular switches by regulating signal transduction pathways for hormones, growth factors and cytokine receptors, including the Raf/MEK/ERK (MAPK) and PI3-K/Akt kinase cascades, and so affect diverse cellular functions. *KRAS* mutation is predictive of response to panitumumab and cetuximab therapy in colorectal cancer.[14] Currently, the most reliable way to predict response of a colorectal cancer patient to one of the EGFR-inhibiting drugs is to test for certain “activating” mutations in the gene that encodes *KRAS*—a protein that transmits growth signals from EGFR—which occur in 30-50% of colorectal cancers.

Studies show patients whose tumors express the mutated version of the *KRAS* gene do not respond to Erbitux or Vectibix; however, some CRC patients with the wild-type (normal) *KRAS* gene also do not respond and PIK3CA mutations are the cause for resistance in this subgroup of patient.[15] Oncogenic mutations in the Ras gene are present in approximately 30% of all human cancers. *KRAS* mutations occur frequently in non-small-cell lung, colorectal, and pancreatic carcinomas.[16] Earlier studies in human colorectal cancers have mainly focused on the frequencies and specific types of point mutations in the *KRAS* oncogene in colorectal cancer.[7,17–19] In colorectal carcinomas, *KRAS* mutation has been reported within a broad range of 30 to 50%.[13,17,20,21] This broad range of reported frequencies of *KRAS* mutations could be due to various factors such as the sensitivity and specificity of mutation detection techniques, patient sample size, variability in analyzed gene region, i.e. only codons 12,[22] 13[23] and/or 61[24] and/or environmental factors. Earlier reports have shown similar propensity of *KRAS* mutations to be located in exon 12.[25,26]

Sameer *et al.* also explored *KRAS* mutational status in the cohort of Kashmiri CRC and found that all identified mutations were missense and most led to the substitution of glycine for aspartate.[8] Their *KRAS* mutation frequency was 22.64: 61.5% in codon 12 and 38.5% in codon 13. Based on these low frequencies, Sameer and colleagues suggest that the etiological factors in Kashmiri CRC are likely to be different and hypothesize that, *KRAS* mutation may not be a common early event in carcinogenesis.[8] The predominance of codon 12 and 13 mutations was expected as most of the mutations found in *KRAS* in human tumors involve these two codons that code for the two adjacent glycine residues that play an important role in the catalytic site of RAS protein. Furthermore, the rate of transitions (84.6%) was found to be higher than that of the transversions (15.4%) in agreement with earlier studies. All the transitions were of the G → A type, affecting the second base of codon 12 (GGT > GAT) in seven patients, first base of codon 12 (GGT > GAT) in one patient, and second base of codon 13 (GGC > GAC) in three patients.

Almost all information on the molecular features of human malignancies is derived from European and US patients. There is, however, growing evidence that these findings may not be applicable to all ethnic groups.[27] For example, genetic differences in the pattern of *TP53* mutations have been reported between Midwest US Caucasian, African-American, Austrian, and Japanese breast cancer patients.[28]

In a similar endeavor to explore ethnic differences in colon cancer if any we had earlier studied potential genetic differences between colon cancers from Saudi Arabia and Western countries.[29] Fluorescence *in situ* hybridization analysis (FISH) was employed in a tissue microarray cohort of 518 colon cancers from Saudi Arabia and Switzerland to estimate frequencies of copy number changes of known oncogenes, including HER2, TOPO2A, CCND1, EGFR, and CMYC. Although no molecular differences were found between Swiss and Saudi colon cancers, rare high-level amplifications of therapeutic target genes were found in colon cancer. Our data does not suggest major molecular differences between Saudi and Swiss colon cancers, but these observations emphasize the urgent need for clinical studies investigating the effect of targeted therapies. In the era of targeted tumor therapies, these observations emphasize the urgent need for clinical studies investigating the effect of targeted therapies with an established patient benefit in one tumor type also in other tumor with similar molecular features.

Considering the smaller cohort size (n = 53), this pilot study by Sameer *et al.* needs to be followed up in a bigger cohort of patients to study the incidence and further characterize common genetic mutations in Kashmiri CRC.[8] Treatment of colorectal carcinoma will evolve with the strides made in molecular diagnostics and targeted therapy. Commonly occurring oncogenes such as *TP53* and *KRAS* should be meticulously studied in populations where there is paucity of data on mutations, to learn more about the molecular signature of the tumors and ethnic differences if any. An emphasis on translational research, better cooperation between oncologist, surgeons, pathologist and researchers, meticulous harvesting and bio-banking of precious tumor tissue material are some key areas that need to be dramatically improved.

Similar studies and continued “dissection of the molecular genome” will unravel the genetic signature and hopefully pave a way for potent but highly selective therapy, tailored for each cancer patient.
